# Anesthesia Management of Hip Fracture Surgery in Geriatric Patients: A Review

**DOI:** 10.7759/cureus.70188

**Published:** 2024-09-25

**Authors:** Tarun Uppalapati, Imani Thornton

**Affiliations:** 1 Anesthesiology, HCA Florida Westside Hospital, Plantation, USA; 2 Anesthesiology and Critical Care, HCA Florida Westside Hospital, Plantation, USA

**Keywords:** anesthesia management, elderly hip fracture, geriatric anesthesia, geriatric hip fracture, orthopedic anesthesia

## Abstract

Hip fractures are increasingly prominent concerns in healthcare, especially in light of a growing elderly population. These fractures contribute to mortality and morbidity in the elderly. Many hip fractures require emergent surgical intervention and may have consequences of serious postoperative complications. Multidisciplinary approaches of management have been utilized to optimize care and improve patient outcomes. Due to the myriad of multi-organ system comorbidities that are increasingly prevalent in the geriatric population, anesthetic management proves to be difficult to standardize. Enhanced Recovery After Surgery (ERAS) protocols have shown benefits for optimizing patient outcomes by focusing on premedication, nutritional support, and pain management; however, specific anesthetic management techniques are not discussed. This literature review aims to discuss ways to optimize hip fracture management in geriatric patients through effective preoperative evaluation, anesthetic considerations, and postoperative optimization and care. A review of currently available guidelines may help determine the optimal management for hip fractures in geriatric patients and improve immediate and lasting postoperative outcomes.

## Introduction and background

Hip fractures are a prevalent form of injury that occur in over 1.6 million individuals worldwide and are expected to reach more than six million by 2050 [1,2]. Hip fractures have remained a key concern in healthcare over the past several years, as they are one of the most common reasons for elderly patients to present to the emergency department (ED) and require urgent surgery. Hip fractures can be associated with poor outcomes in the elderly population [3]. Over 90% of all hip fractures occur in patients 65 years and older and have various comorbidities [4]. Management of elderly patients proves challenging, especially in those patients with cardiopulmonary or cerebral dysfunction [5]. These added risks lead to higher rates of perioperative morbidity and mortality within the elderly population. Moreover, geriatric patients and those with end-stage renal disease on dialysis often have higher incidences of morbidity and mortality following hip fracture surgeries [6].

With an increasingly aging population, it is important to consider appropriate anesthetic management and outcomes of geriatric patients following fragility fractures. Studies have shown the benefits of having an integrated approach with a multidisciplinary team to ensure effective analgesia, early mobilization, and ultimate improvement in patient outcomes [7]. The current models of anesthetic use for hip fracture surgery are opioids, neuraxial and general anesthesia, regional nerve blocks, and mobilization on the day after surgery [7]. However, the most optimal anesthesia technique remains controversial across various studies. This paper reviews various retrospective and prospective trials to elucidate best practices to optimize patient safety and perioperative outcomes in this patient population (Table 1).

**Table 1:** Summary of articles PENG: pericapsular nerve group; CKD: chronic kidney disease; ACC: American College of Cardiology; AHA: American Heart Association; ERAS: Enhanced Recovery After Surgery; THA: total hip arthroplasty; TKA: total knee arthroplasty; COX-2: cyclooxygenase-2; TNS: transient neurological symptoms; FICB: fascia iliaca compartment block; CFNC: continuous femoral nerve catheter; NSAIDs: nonsteroidal anti-inflammatory drugs; CHF: chronic heart failure; COPD: chronic obstructive pulmonary disease; ASA: American Society of Anesthesiologists

Authors	Aim of study	Type of study/information	Conclusion
Dhanwal et al., 2011 [1]	Describe the incidence of hip fracture in different regions of the world	Review	The highest hip fracture rates are seen in North Europe and the United States.
Zheng et al., 2020 [2]	Compare the effect of neuraxial (epidural/spinal) versus general anesthesia on perioperative outcomes in patients undergoing hip fracture surgery	Meta-analysis	There may be a difference in blood loss between patients receiving neuraxial and general anesthesia; however, the study is at high risk for type 1 error.
Turner, 2021 [3]	Information on perioperative medical co-management of patients with geriatric hip fractures	Chapter in a book	Since fracture repair is recommended within 48 hours for mortality benefit, most testing should not delay the operation.
Carpintero et al., 2014 [4]	Complications of hip fractures	Review	Over 90% of hip fracture patients are older than 65 years old and have preexisting medical comorbidities. Elderly trauma patients suffer higher morbidity and mortality rates when compared to the general population.
Niu et al., 2022 [5]	Anesthetic management of hip fracture in a geriatric patient with respiratory and heart failure using PENG block	Case report	PENG block combined with hip nerve blocks resulted in a stable hemodynamic process, a satisfactory anesthetic effect, and an effective postoperative analgesia and had no effect on postoperative cognitive function.
Yoon and Koo, 2017 [6]	Hip fracture in CKD patients	Review	One-year mortality of CKD patients who sustain hip fracture is up to 70%, and dialysis patients had a 13.7 greater chance of death at one year post-surgery compared to age- and sex-matched non-dialysis patients. A comprehensive treatment approach reduces morbidity and mortality of hip fractures in CKD patients.
Maxwell and White, 2013 [7]	Anesthetic management of patients with hip fractures	Review	Evidence for anesthetic intervention remains poor, and a multidisciplinary national research program will help identify which outcomes are most appropriate as study endpoints and which treatments are most effective.
Alvi et al., 2018 [8]	Complications and outcomes in elderly hip fracture surgery as a function of time to surgery	Cohort	Time to surgery greater than 48 hours is associated with a costly increase in the total length of stay, including an increased post-surgery-to-discharge time.
Pincus et al., 2017 [9]	Association between wait time and 30-day mortality in patients undergoing hip fracture surgery	Cohort	Among adults undergoing hip fracture surgery, increased wait time was associated with a greater risk of 30-day mortality and other complications. A wait time of 24 hours may represent a threshold defining higher risk.
Mitchell et al., 2018 [10]	Evaluate the association between the timing of hip fracture surgery and postoperative length of stay and outcomes	Cohort	Although a delay in the management of hip fractures is associated with an increase in postoperative hospital length of stay, 30-day postoperative outcomes are not adversely affected in patients undergoing hip fracture fixation.
Smeets et al., 2020 [11]	Investigate the adherence to the ACC and AHA guidelines for the perioperative assessment of patients with hip fractures in daily clinical practice and how this might affect outcome	Cohort	Preoperative cardiac screening for hip fracture patients in adherence to ACC and AHA guidelines is associated with diminished use of preoperative resources. Over-screening leads to greater delay in surgery, which poses a risk for perioperative complications and early mortality.
Fleisher et al., 2014 [12]	ACC and AHA guidelines for patients undergoing noncardiac surgery	Review	Multiple guidelines on the preoperative, perioperative, and postoperative care of patients undergoing noncardiac surgery.
Chen et al., 2019 [13]	Effect of frailty and relationship with one-, three-, and six-month postoperative emergency department visits, readmissions, and mortality	Cohort	Frailty has a long-term effect on each adverse outcome after discharge, rather than the effect simultaneously. Targeting pre-frailty and frailty is essential to prevent adverse outcomes and improve the overall health of older adults after discharge from a hip fracture.
Kua et al., 2016 [14]	Discover which frailty measure is more suitable for the prediction of early postoperative outcomes in hip fracture patients	Cohort	Frailty, measured by the modified Fried criteria and Reported Edmonton Frail Scale, is a good predictor of early postoperative outcomes in older adults undergoing hip surgery. It can also predict six months of basic activities of daily living function.
Lin et al., 2016 [15]	Examine the impact of frailty on adverse outcomes in the "older-old" (75-85-year-old) and "oldest-old" (over 85-year-old) surgical candidates	Review	There was strong evidence that frailty in older-old and oldest-old surgical patients predicts postoperative mortality, complications, and prolonged length of stay.
Ma et al., 2022 [16]	Evaluate the association between preoperative frailty and postoperative outcomes in patients with hip fractures	Meta-analysis	Preoperative frailty may be associated with postoperative adverse events in patients with hip fractures, including in-hospital mortality, 30-day mortality, and postoperative complications.
Shimizu et al., 2022 [17]	Investigate the association between frailty and adverse events in patients with hip fractures after surgery	Meta-analysis	Frailty risk was defined using the Hospital Frailty Risk Score and was able to predict adverse events, including in-hospital mortality, in Japanese older patients with hip fractures.
Ha et al., 2021 [18]	Assess the prevalence of dementia as an underlying disease in elderly patients with hip fractures and analyze the differences in postoperative mortalities according to the severity of dementia	Case-control	In elderly hip fracture patients, the comparison between patients with and without dementia revealed that dementia was an independent risk factor for mortality at a minimum of one year of follow-up and severity of dementia in hip fracture patients was a risk factor for mortality within six months and one year, postoperatively.
Petersen et al., 2020 [19]	Investigate clinical management including time to surgery, out-of-hours admission and surgery, surgery on weekends, surgery volume per ward, and anesthesia technique for this excess mortality risk	Cohort	Overall postoperative 30-day mortality in patients with dementia had a 1.5 times increased mortality risk than those without dementia.
Ljungqvist et al., 2017 [20]	Review of a multimodal, multidisciplinary approach to the care of the surgical patient	Review	ERAS is an evidence-based care improvement process for surgical patients. Implementation of this program results in major improvements in clinical outcomes and cost.
Choi et al., 2022 [21]	Summarize the components of the ERAS protocol applied to orthopedic surgery, evaluate the outcomes of ERAS, and suggest practical strategies to implement the ERAS protocol successfully	Review	ERAS pathway has shortened the length of stay, reduced postoperative complications, and improved patient outcomes and satisfaction.
Wainwright et al., 2020 [22]	Summarize the literature and propose recommendations for the perioperative care of patients undergoing total hip replacement and total knee replacement with an ERAS program	Review	Best practice includes preoperative patient education, anesthetic technique, and transfusion strategy, in combination with an opioid-sparing multimodal analgesic approach and early mobilization.
Ripollés-Melchor et al., 2020 [23]	Assess the association of the use of ERAS protocols with complications in patients undergoing elective THA and TKA	Review	An increase in adherence to the ERAS program was associated with a decrease in postoperative complications, although only a few ERAS items were individually associated with improved outcomes.
Volkow et al., 2011 [24]	Analyze the prescription practices in the United States to identify possible contributors to the high rate of opioid analgesic abuse	Cohort	On average, across all physician specialties included in this analysis, 56.4% of opioid prescriptions were dispensed to patients who had already filled another opioid prescription within the past month.
Anciano Granadillo et al., 2018 [25]	Examine the trends in preoperative and prolonged postoperative opioid analgesic use in patients undergoing hip arthroscopy, characterize the risk factors for prolonged opioid analgesic use following hip arthroscopy, and explore preoperative and prolonged postoperative opioid analgesic use as independent risk factors for complications following hip arthroscopy	Cohort	The most significant risk factor for prolonged opioid use is preoperative opioid analgesic use. Preoperative and prolonged postoperative opioid analgesic use was associated with a higher likelihood of several adverse effects/complications.
Cepeda et al., 2003 [26]	Evaluate the risk factors that increase the risk of developing opioid side effects	Cohort	The risk of respiratory depression increases substantially after 60 years of age.
Thijssen et al., 2022 [27]	Evaluate the differences in perioperative multimodal pain management strategies for THA and TKA in the Netherlands and study the associations between patient- and therapy-related factors and pain outcomes	Cohort	Although no ideal strategy was identified, the use of NSAIDs, ketamine, and dexamethasone was associated with less pain and less side effects.
Anger et al., 2021 [28]	Develop recommendations for the management of postoperative pain after primary elective THA, updating the previous procedure-specific postoperative pain management guidelines	Review	Multiple guidelines for pain control to facilitate early postoperative rehabilitation, which is being increasingly encouraged in recent guidelines.
Jiang et al., 2020 [29]	Evaluate the efficacy and safety of selective COX-2 inhibitors in postoperative pain management in patients receiving THA/TKA	Meta-analysis	Selective COX-2 inhibitor therapy is effective, safe, and reliable in relieving postoperative pain of THA/TKA.
Fillingham et al., 2020 [30]	Evaluate the efficacy and safety of acetaminophen in support of the combined clinical practice guidelines of the American Association of Hip and Knee Surgeons, American Academy of Orthopedic Surgeons, Hip Society, Knee Society, and American Society of Regional Anesthesia and Pain Management	Meta-analysis	Strong evidence supports the safety of oral and intravenous acetaminophen when appropriately administered to patients undergoing primary total joint arthroplasty. Moderate evidence supports the use of oral and intravenous acetaminophen as a non-opioid adjunct for pain management during inpatient hospitalization.
Smith et al., 2023 [31]	Explain the role of general anesthesia and outline relative contraindications to general anesthesia	Education article	Choosing the available anesthetics for patient safety for general anesthesia depends on patient factors including patient characteristics, surgeon/anesthesiologist preference, and type of surgery being performed.
Olawin et al., 2022 [32]	Explain the role of spinal anesthesia and outline indications and complications of spinal anesthesia	Education article	Neuraxial anesthesia use depends on patient factors, the post-op team needs to be aware of the procedure, and the patient needs to be monitored by well-trained personnel.
Mauermann et al., 2006 [33]	Test the hypothesis that elective total hip replacement under neuraxial block was associated with improved outcomes compared with the surgery under general anesthesia	Meta-analysis	Patients undergoing elective total hip replacement under neuraxial anesthesia seem to have better outcomes than those under general anesthesia.
Knio et al., 2023 [34]	Assess the effect of spinal versus general anesthesia on healthcare resource utilization and secondary endpoints following THA	Cohort	THA patients receiving spinal anesthesia experience favorable outcomes compared to propensity-matched general anesthesia patients.
Desai et al., 2018 [35]	Measure if 90-day mortality differs depending on the type of anesthesia received and if 90-day readmissions differ based on anesthesia technique after geriatric hip fracture repairs	Cohort	General anesthesia is associated with a higher risk of all-cause readmission compared with regional, but no other differences were observed in the risk for complications. Regional techniques may be preferred, when possible, in geriatric patients.
Neuman et al., 2023 [36]	Understand the benefits and harms of spinal versus general anesthesia for surgery in older adults undergoing hip fracture surgery, elective knee and hip arthroplasty, and vascular surgery	Review	Spinal and general anesthesia are likely equivalent in terms of safety and acceptability for most patients without contraindications. The decision between spinal and general anesthesia depends on patients' preferences and values.
Guo et al., 2022 [37]	Evaluate the effect of anesthesia on postoperative complications in older patients undergoing hip surgery	Cohort	Results showed no difference in postoperative complications between elderly patients undergoing hip surgery under spinal anesthesia and general anesthesia.
Khan et al., 2021 [38]	Compare the early postoperative mortality between general and regional anesthesia administered to patients undergoing either THA or partial hip arthroplasty	Cohort	There is no association between general and regional anesthesia and early postoperative mortality rates in patients undergoing hip arthroplasty. Early postoperative mortality is low in the absence of risk factors such as severe CHF, COPD, ascites, acute renal failure, and an ASA score of 4 or greater.
Neuman et al., 2021 [39]	Examine the effects of spinal anesthesia as compared with general anesthesia on the ability to walk in older adults undergoing surgery for hip fracture	Randomized controlled trial	Spinal anesthesia for hip fracture surgery in older adults was not superior to general anesthesia with respect to survival and recovery of ambulation at 60 days. The incidence of postoperative delirium was similar between the two types of anesthesia.
Herndon et al., 2021 [40]	Determine what, if any, effect the dose of bupivacaine spinal anesthesia had on perioperative outcomes in total joint arthroplasty	Cohort	The dosage of bupivacaine spinal anesthetic did not affect perioperative outcomes.
Slaven et al., 2023 [41]	Study the comparative rates of TNS and postoperative urinary retention between lidocaine, mepivacaine, and bupivacaine in a high-volume hip and knee arthroplasty setting	Cohort	No significant difference in TNS rates among lidocaine, mepivacaine, and bupivacaine. The rate of urinary retention requiring catheterization was higher with bupivacaine than with mepivacaine and lidocaine.
Calkins et al., 2021 [42]	Compare the recovery, pain, and complications between mepivacaine and bupivacaine in THA at an ambulatory surgery center	Cohort	Mepivacaine spinal anesthesia for THA safely facilitated more rapid same-day discharge due to decreased time to controlled void and ambulation with only a minor increase in pain when compared to bupivacaine.
Herndon et al., 2020 [43]	Compare the perioperative outcomes of patients undergoing fast-track THA using chloroprocaine spinal anesthesia with those who have surgery with a longer-acting agent, bupivacaine	Cohort	Chloroprocaine is a safe and reliable option for patients to mobilize rapidly and leave the hospital sooner after THA. Compared to bupivacaine, it is associated with shorter hospital length of stay and a higher likelihood of discharge to home.
Husted et al., 2010 [44]	Report the results with short-duration pharmacological prophylaxis combined with early mobilization and reduced hospitalizations	Cohort	Risk of clinical deep vein thrombosis and of fatal and non-fatal pulmonary embolus after THA and TKA following a fast-track set-up with early mobilization, short hospitalization, and short duration (6-8 hours after surgery until discharge) of deep vein thrombosis prophylaxis compares favorably with published regimens with extended prophylaxis (up to 36 days) and hospitalization up to 11 days.
Andreasen et al., 2017 [45]	Present the baseline detailed economical calculations of fast-track THA and TKA and compare these between the two departments	Cohort	Compared to the cost associated with longer more conventional published pathways, fast-track is cheaper, which adds to cost efficiency and the potential for economic savings.
Frisch et al., 2018 [46]	Determine the safety and efficacy of single-dose lidocaine spinal anesthesia in the setting of outpatient joint arthroplasty	Cohort	Isobaric lidocaine spinal anesthesia is a safe and effective regimen for outpatient hip and knee arthroplasty, and patients were discharged on the day of surgery with no reports of TNS.
Stambough et al., 2019 [47]	Compare the spinal and general anesthesia for rapid recovery after total joint arthroplasty	Case series	Modern general anesthesia techniques that limit narcotics and certain inhalants can be successfully used in short-stay primary total joint arthroplasty.
Zhang et al., 2020 [48]	Investigate the effects of dexmedetomidine on postoperative delirium and pro-inflammatory markers in elderly patients with hip fracture	Randomized controlled trial	Dexmedetomidine reduced the incidence of postoperative delirium in elderly patients on the first day after hip fracture surgery and reduced the inflammatory markers over the first three days after surgery.
Dangle et al., 2020 [49]	Describe the anatomical and technical aspects of various regional techniques used for hip fracture and hip surgery	Review	Complementing with regional anesthesia in the elderly population undergoing hip surgery has shown numerous benefits.
Maniar et al., 2021 [50]	Describe the relevant anatomy, technical approach, associated risk, and practicability to facilitate a better understanding of the various approaches available	Review	The effectiveness of nerve blockade over pharmacological therapy is established. No single peripheral nerve block has a significant advantage over other types.
Verbeek et al., 2021 [51]	Review the perioperative effects of the FICB in hip fracture patients	Review	FICB reduces opioid consumption, decreases morbidity and mortality, reduces hospital stay, reduces delirium, and improves satisfaction.
Li et al., 2022 [52]	Compare the femoral nerve block and FICB for proximal femoral fracture in the elderly patient	Meta-analysis	FICB had a reduction in the Visual Analog Pain Scale, while the femoral nerve block decreases the risk of several adverse events.
Luo et al., 2022 [53]	Test the hypothesis that PENG block can enhance the recovery in elderly patients after hip fracture surgery	Randomized controlled trial	This nerve block was seen to relieve pain without motor dysfunction to accelerate recovery.
Allard et al., 2021 [54]	Compare the efficacy and side effects of recently described PENG block with those of femoral block, which is considered the standard of care for postoperative pain control after a femoral neck fracture	Observational	PENG block did not have a significant change in postoperative morphine consumption, compared to femoral block. It does however significantly improve immediate mobility of the operated limb.
Mosaffa et al., 2022 [55]	Evaluate the PENG block in control of hip fracture pain and compare it with FICB	Randomized controlled trial	PENG block is a good method in hip fracture analgesia and provides better analgesia than FICB.
Vamshi et al., 2023 [56]	Compare the efficacy of the PENG block versus suprainguinal fascia iliaca block in THA	Randomized controlled trial	PENG block significantly reduces perioperative morphine consumption and pain scores in THA patients when compared to the suprainguinal fascia iliaca block.
Rasappan et al., 2021 [57]	Compare the efficacy of the continuous infusion FICB on perioperative pain relief, opioid usage, its associated complications, and the short- as well as long-term rehabilitation status in geriatric hip fracture patients	Case-control	Continuous infusion FICB provides safe and effective postoperative pain relief in geriatric hip fracture patients. Postoperative opioid usage is decreased in older hip fracture patients treated with this infusion. Rehabilitation milestones are slower in the short term, but there is no significant difference at one year post-surgery.
Arsoy et al., 2017 [58]	Examine the effect of perioperative CFNC blockade with regard to pain scores, opioid-related side effects, and posthospital disposition in hip fracture patients undergoing THA	Cohort	CFNC blockade patients were discharged home more frequently. It also decreased daily average patient-reported pain scores, preoperative opioid usage, and opioid-related side effects after THA for hip fracture.
Wu and Han, 2020 [59]	Explore how CFNC block affects the incidence of postoperative cognitive dysfunction among low-risk and high-risk patients with femoral neck fractures	Cohort	Perioperative CFNC block administration is useful in decreasing the incidence of postoperative cognitive dysfunction, especially for high-risk patients with a Mini-Cog score equal to or less than 2 points.

## Review

Preoperative evaluation

In patients with hip fractures, a surgery delay of >48 hours significantly prolongs the time from surgery to discharge [8]. Pincus et al. identified that a wait time of 24 hours may represent a better threshold for defining a higher risk of complications that may result from delays in surgical intervention [9]. Mitchel et al. found that increased time to surgery is correlated with increased postoperative length of stay [10]. Therefore, patients who have hip fractures may possibly benefit from being prioritized and balanced with other patients on the same-day surgery list.

Preoperative cardiac consultation can be time-consuming and may lead to delays in surgery [10]. It is important to consider whether a cardiac consultation will change an anesthetic plan. Smeets et al. divided patients in a prospective study into low, intermediate, and high risk for cardiac complications. In the study, they observed that cardiac over-screening occurred in patients of the intermediate-risk group [11]. Intermediate risk classification may be more difficult to categorize for patients without obvious low- or high-risk comorbidities. The American College of Cardiology (ACC) and American Heart Association (AHA) guidelines require non-invasive cardiac tests in active high-risk cardiac patients and in intermediate-risk patients based on functional capacity, which is measured by a metabolic equivalent of task [12]. Strict adherence to these guidelines may help to prevent unnecessary cardiac consultations and avoid delays to surgical intervention.

Additional preoperative considerations in geriatric patients include dementia and frailty, since these factors can increase the risk of surgical complications. Frailty has been shown to predict adverse outcomes in elderly surgical patients [13-16]. Chen et al. assessed frailty in elderly patients following the surgical treatment of hip fracture and divided it into three categories, robust, pre-frail, and frail, based on the Chinese-Canadian Study of Health and Aging Clinical Frailty Scale [13]. Their findings demonstrated increased mortality rates in frail patients post-discharge compared to their healthy counterparts. Moreover, frail patients were more likely to have early ED visits one month following discharge and readmission within three months of discharge [13]. 

Shimizu et al. assessed the Hospital Frailty Risk Score (HFRS), which uses the International Statistical Classification of Diseases 10th Revision (ICD-10) codes for inpatients, and divided patients with hip fractures aged ≥65 years into three categories: low risk (<5), intermediate risk (5-15), and high risk (>15). The study found that patients with higher frailty risk scores were more likely to present with comorbidities that impaired functional recovery and were associated with poorer functional outcomes.

The incidence of dementia has been increasing in many countries with an aging global population and is a factor that can increase hip fracture risk [18,19]. Ha et al. identified the severity of dementia as a significant predictor of mortality one year postoperatively in hip fracture patients [18]. Petersen et al. assessed elderly patients with first-time hip fractures in 2013-2014 and found that patients with dementia had 1.5 times higher postoperative 30-day mortality compared to patients without dementia. In addition to dementia, the study states that prognostic factors important for 30-day mortality include increased age, male sex, comorbid chronic diseases, taking sedative medications, and having a higher American Society of Anesthesiologists (ASA) class score [19]. Ultimately, effective preoperative assessment is essential to optimize postoperative success. Special preoperative considerations for dementia and frailty are important in the surgical optimization of postoperative hospital stay, morbidity, and mortality. 

Enhanced Recovery After Surgery (ERAS) protocols

The ERAS Society was created in Europe in 2001 to develop a care pathway for patients undergoing colorectal procedures [20]. ERAS guidelines are updated often for a variety of surgical operations. ERAS protocols have resulted in shorter hospital stay by 30-50% in addition to reductions in complications, readmissions, and costs [20].

There are about 17 components of the ERAS protocol recommended for orthopedic surgery. These components are distributed among preoperative, perioperative, and postoperative periods [21]. Wainwright et al. outlined the ERAS recommendations for perioperative care in total hip replacements [22]. Some strong and high evidence-level recommendations mentioned in this article include smoking cessation of four weeks or more, correction of preoperative anemia, use of tranexamic acid (TXA) for the reduction of blood loss, use of nonsteroidal anti-inflammatory drugs (NSAIDs) recommended for patients without contraindications, minimizing the use of opioids, maintaining normothermia perioperatively and postoperatively, and mobilization of patients as early as possible [22].

A multicenter study conducted by Ripolles-Melchor et al., involving 131 centers, observed the adherence to ERAS protocols in elective hip and knee surgeries for adult patients who were followed for up to 30 days after surgery [23]. The severity of postoperative complications 30 days after surgery and length of stay and mortality were all improved in patients who had the highest adherence to ERAS components [23]. Successes with ERAS protocols highlight the benefits to perioperative outcomes in orthopedic surgery patients, although there remain no standard guidelines specific to the management of the elderly hip fracture patients.

Multimodal pain management

Opioids are commonly used for pain control in patients, and orthopedic surgeons are among the top prescribers of opioids [24]. Recent literature currently recommends caution with using opioids due to increasing complications with opioid consumption. Drug overdose deaths in the United States almost tripled between 1999 and 2014 [25]. Anciano Granadillo et al. found that preoperative and prolonged postoperative opioid analgesic use was associated with postoperative complications such as increased ED visits and revision surgery [25]. Cepeda et al. identified a correlation between increased risk of respiratory depression and increased age after 60 years old [26]. As a result, multimodal pain management, which includes two or more different groups of analgesic drugs, can be used to reduce opioid consumption and related side effects [27].

Procedure-Specific Pain Management (PROSPECT) guidelines for total hip arthroplasty, created from a global collaboration of surgeons and anesthesiologists, recommend a combination of acetaminophen and NSAIDs or cyclooxygenase-2 (COX-2) selective inhibitors administered preoperatively and continued postoperatively [28]. Since NSAIDs come with their own set of adverse effects including symptoms of dyspepsia and gastroesophageal reflux, COX-2 selective inhibitors may be a better option. Jiang et al. recently published a meta-analysis of the efficacy and safety of selective COX-2 inhibitors for postoperative pain management in patients after total knee and hip arthroplasties. They found that COX-2 inhibitors decreased opioid use within three days after surgery as well as decreased the incidence of nausea, vomiting, and fever [29].

Acetaminophen is a regularly prescribed medication and is a moderate to strong recommendation for use in patients with hip arthroplasties [22]. Acetaminophen use may mitigate the risk of untoward adverse effects of other medications including gastrointestinal symptoms, altered mental status, and respiratory depression. Fillingham et al. conducted a meta-analysis review to determine the efficacy and safety of acetaminophen in total joint arthroplasty. They found supporting evidence to use it as a non-opioid adjunct for pain management. The authors also found no significant difference in postoperative pain and opioid consumption when comparing oral and intravenous acetaminophen administration [30].

There has been inconsistent evidence regarding the use of gabapentinoids in multimodal pain management regimens. Three meta-analyses that assessed the efficacy of gabapentin or pregabalin in total hip arthroplasty reported side effects such as dizziness and inconsistent findings with pain reduction [28]. These medications are not currently recommended to be adjuncts in multimodal management [22,28]. The PROSPECT trial recommends the use of intravenous dexamethasone 8-10 mg for its analgesic and anti-emetic effects [28]. Thijssen et al. followed postoperative pain scores following total joint arthroplasties and found that the use of dexamethasone was independently associated with less pain in patients [27]. NSAIDs, COX-2 inhibitors, acetaminophen, and dexamethasone can all be considered for multimodal pain management based on currently available literature. The use of a combination of these medications may prove useful in avoiding the undesirable adverse effects of opioids in the elderly population undergoing hip fracture surgeries.

Anesthetic techniques

The two primary considerations for anesthesia technique in this patient population are general and neuraxial (spinal) anesthesia. There is no current consensus on which is superior to maximize patient safety and perioperative outcomes. 

While some studies stated that spinal anesthesia is possibly superior to general anesthesia for older patients [33,34], others have found conflicting results when comparing the two. Desai et al. noted that general anesthesia is associated with a higher risk of mortality and readmission within 90 days of surgery in geriatric patients when compared with spinal anesthesia [35]. In contrast, Neuman et al.'s review concluded that both neuraxial and general anesthetics yielded comparable outcomes across several endpoints, inducing delirium, length of stay, mortality, and functional recovery.

Other researchers have also found no significant difference between both techniques. Guo et al. looked at 309 patients to identify a relation between anesthetic technique and complications after hip surgery in the elderly population and found that 67 of those patients had complications including limb dysfunction, pulmonary infection, delirium, lower extremity venous thrombosis, and shock [37]. The incidence of these complications in this study was not related to the anesthetic technique used. Khan et al. looked at a total of 60,897 patients and observed that there is no association between the type of anesthesia and early postoperative mortality rates in patients undergoing hip arthroplasty [38]. Neuman et al. randomly assigned patients to have either spinal or general anesthesia to observe the primary outcomes of death or inability to walk 10 feet independently or with a walker/cane at 60 days after surgery [39]. They had a total of 1600 patients enrolled and concluded that spinal anesthesia for hip fracture surgery in older adults was not superior to general anesthesia with regard to survival and recovery of ambulation at 60 days. With the conflicting outcomes in the available literature, there remains no consensus as to which anesthetic technique is superior in this patient population. Anesthetic management of these patients warrants an individualized risk versus benefit consideration to determine which anesthetic technique may be more beneficial for a particular patient. In instances where spinal anesthesia is absolutely contraindicated (lack of patient consent, elevated intracranial pressure due to intracranial mass, and infection at the site of the procedure), general anesthesia may be the only available option [32].

Spinal Anesthesia

A common choice of agent to use for spinal anesthesia in hip arthroplasty is bupivacaine [40-43]. Bupivacaine is known to be cost-effective and has a safe side-effect profile. On the other hand, bupivacaine's long duration of action can increase the risk of urinary retention and prolonged hospitalization. The overall benefits may be more desirable in the instance of "fast-track" total hip arthroplasty, which has been shown to result in early mobilization, shorter hospitalization, and less postoperative mortality [44,45]. "Fast-track" programs involve a multidisciplinary team that optimizes logistics including homogenous wards and provide extensive preoperative patient preparatory materials to ease throughout [44]. Imperative to spinal use in fast-track protocols are shorter-acting and more predictable local anesthetic options. In a retrospective study by Herndon et al., decreased doses of isobaric bupivacaine (<15 mg) were compared to 15 mg of isobaric bupivacaine and found no difference in outcomes including length of stay [40]. Shorter-acting anesthetic agents such as mepivacaine, lidocaine, and chloroprocaine may also provide a reasonable alternative to the longer duration of action of bupivacaine. Mepivacaine and lidocaine were previously reported to cause incidences of transient neurologic symptoms (TNS) and had fallen out of favor for use in neuraxial anesthesia. Recently, Slaven et al. did a retrospective study with 1,217 patients undergoing hip and knee arthroplasty to compare the rates of TNS and urinary retention in spinal anesthesia between lidocaine, mepivacaine, and bupivacaine [41]. The study found no difference in the rate of TNS between the three agents, as well as a lower incidence of postoperative urinary retention with the shorter-acting lidocaine and mepivacaine. Calkins et al. observed that mepivacaine spinal anesthetic caused more rapid same-day discharge and decreased time to controlled void and ambulation when compared to bupivacaine [42]. In a prospective study of patients undergoing hip and knee arthroplasty, Frisch et al. observed that isobaric lidocaine allowed for same-day surgery discharge and found no reports of TNS [46]. Chloroprocaine, a short-acting local anesthetic which has not been extensively studied in this patient population, was recently compared to bupivacaine by Herndon et al. This study also found a shorter length of hospital stay with chloroprocaine [43].

General Anesthesia

General anesthesia is favorable at times and necessary when spinal anesthesia is contraindicated. Stambough et al. did a retrospective study investigating a protocol for general anesthesia that can decrease complications, readmissions, or prolonged length of stay in patients undergoing total joint arthroplasty [47]. Modern general anesthesia techniques that employ ERAS protocols, multimodal pain management, and decreased use of opioids were shown to decrease overall length of stay. In the elderly patient population, general anesthesia may be associated with postoperative cognitive defects including postoperative delirium. Avoidance of this complication, particularly in hip fracture patients, is an important part of decreasing overall morbidity and mortality. Dexmedetomidine, an alpha-2 agonist used for sedation, has shown promise in reducing postoperative delirium, especially when added to general anesthesia regimens. Zhang et al. found that administering dexmedetomidine at 0.5 mcg/kg/hr 30 minutes before the start of anesthesia, continuing the infusion at 0.3 mcg/kg/hr throughout surgery, and discontinuing it 30 minutes before the end of surgery may decrease the incidence of postoperative delirium in elderly patients [48].

Peripheral nerve blocks

Regional anesthesia has become a more prevalent anesthetic technique in recent years among anesthesiologists. Peripheral nerve blocks may be used as part of a multimodal pain regimen, which may decrease opioid use, particularly in the elderly population. In hip fracture patients, commonly employed single-shot nerve blocks include the fascia iliaca compartment block (FICB), femoral nerve block, and pericapsular nerve group (PENG) block. The FICB often employs a large volume of dilute local anesthetic deposited deep into the fascia iliaca, which can spread to the femoral, obturator, and lateral femoral cutaneous nerves [49]. The femoral nerve block targets the femoral nerve, which supplies sensory innervation to the hip joint and both motor and sensory supply to the thigh [49]. The PENG block targets articular branches of the femoral, obturator, and accessory obturator nerves [49]. PENG blocks are gaining popularity for use in these patients as it does not cause significant motor weakness, which allows for early mobilization after hip surgery [49,50].

The main advantage of the FICB is its relatively distal insertion from critical vascular structures like the femoral artery and vein [50]. Verbeek et al. discussed the perioperative effects of FICBs in hip fracture patients in their review article, in which they compared the relevant FICB literature published at that time [51]. Relevant studies showed improved outcomes with FICBs across various domains: reduction in opioid consumption, decrease in morbidity and mortality, shorter hospital stay, reduced delirium, and improved patient satisfaction. Li et al. compared FICB and femoral nerve block outcomes and found that FICB had better postoperative analgesia at four hours, but femoral nerve blocks decreased nausea, vomiting, and sedation [52]. They also found no significant difference with regard to narcotic requirements at 24 hours, mean arterial blood pressures, and patient satisfaction.

The demand for regional anesthetics that minimize motor weakness has led to the increased usage of the PENG block for hip surgery. Many studies indicated that PENG blocks could provide analgesia with minimal to no opioid requirements at 24 hours postoperatively [53]. Allard et al. observed that when compared to femoral nerve blocks, there was significant improvement in immediate mobility of the operative limb with the PENG block [54]. There was also no difference in postoperative morphine consumption between the two blocks. Mosaffa et al. did a randomized controlled trial to compare PENG blocks and FICBs and found that PENG blocks provide better analgesia than FICBs [55]. Furthermore, Vamshi et al. compared both PENG blocks and FICBs and found that PENG blocks significantly reduced morphine consumption and pain scores when compared to FICBs [56]. In this study, they also found that PENG blocks were not associated with quadriceps weakness as was seen in FICBs. To date, PENG blocks have proven to be a valuable addition to regional anesthesia options for the management of pain in hip fracture patients.

Continuous infusion by catheter may provide pain control for longer periods than single-shot injections in some cases. Although they are more often used for arthroplasty patients, hip fracture patients may also benefit. Rasappan et al. observed the use of fascia iliaca compartment catheters in the geriatric population and found that they provided safe and effective pain relief while decreasing postoperative opioid usage [57]. In other studies, continuous femoral nerve catheters were associated with decreased average pain scores, shorter lengths of stay, and decreased incidence of postoperative cognitive dysfunction [58,59]. In circumstances that may require prolonged regional pain management, catheters may provide many of the same benefits as aforementioned for single-shot nerve blocks.

Limitations

The limitations in this review are related to the included articles. One of the limitations is that many of the mentioned articles did not have a control group. For example, when considering multimodal pain management, studies did not have a control group to further assess the effectiveness of interventions. The same is true when comparing different peripheral nerve blocks. Future studies should involve a control group when assessing pain management. Another limitation is that different institutions may have different protocols and standards to the management of care. For example, ERAS protocols can vary from institution to institution. This can cause a variation in the perceived pain control in patients due to multiple different variables that are not controlled. Also, as regional nerve blocks are becoming increasingly popular, there may be a benefit to exploring combinations of nerve blocks. In many of the studies, different individual nerve blocks were compared with each other. It may be possible that doing two different nerve blocks in the same patient may add a combined benefit as opposed to one.

## Conclusions

Hip fractures remain a major area of concern, especially in the geriatric population. While ERAS protocols and multimodal pain management have improved morbidity associated with surgery, no single anesthetic technique has proven superior with regard to outcomes after surgery. Time from injury to surgery has been reported as an important metric for optimal care. Minimizing cardiac over-screening while tailoring preoperative clearance requirements to the patients' comorbid conditions can help to decrease barriers to reach "time to OR" goals. Although there remains a debate on the ideal anesthetic technique for this patient population, there are various aspects to consider in each technique with the primary goals of maximizing patient comfort and minimizing perioperative complications. Peripheral nerve blocks, with or without continuous catheters, have emerged as valuable adjuvants as they may decrease the need for opioids in the perioperative period. Future studies in this area should focus on expanding our understanding of geriatric medicine to facilitate the preoperative, intraoperative, and postoperative customization of anesthetic management in this patient population.
